# Supplementary figures and images for: Mammography screening: Eliciting the voices of informed citizens
Source: PLoS One. 2025 Jan 9;20(1):e0317263. doi: 10.1371/journal.pone.0317263 (PMC11717236; doi:10.1371/journal.pone.0317263)

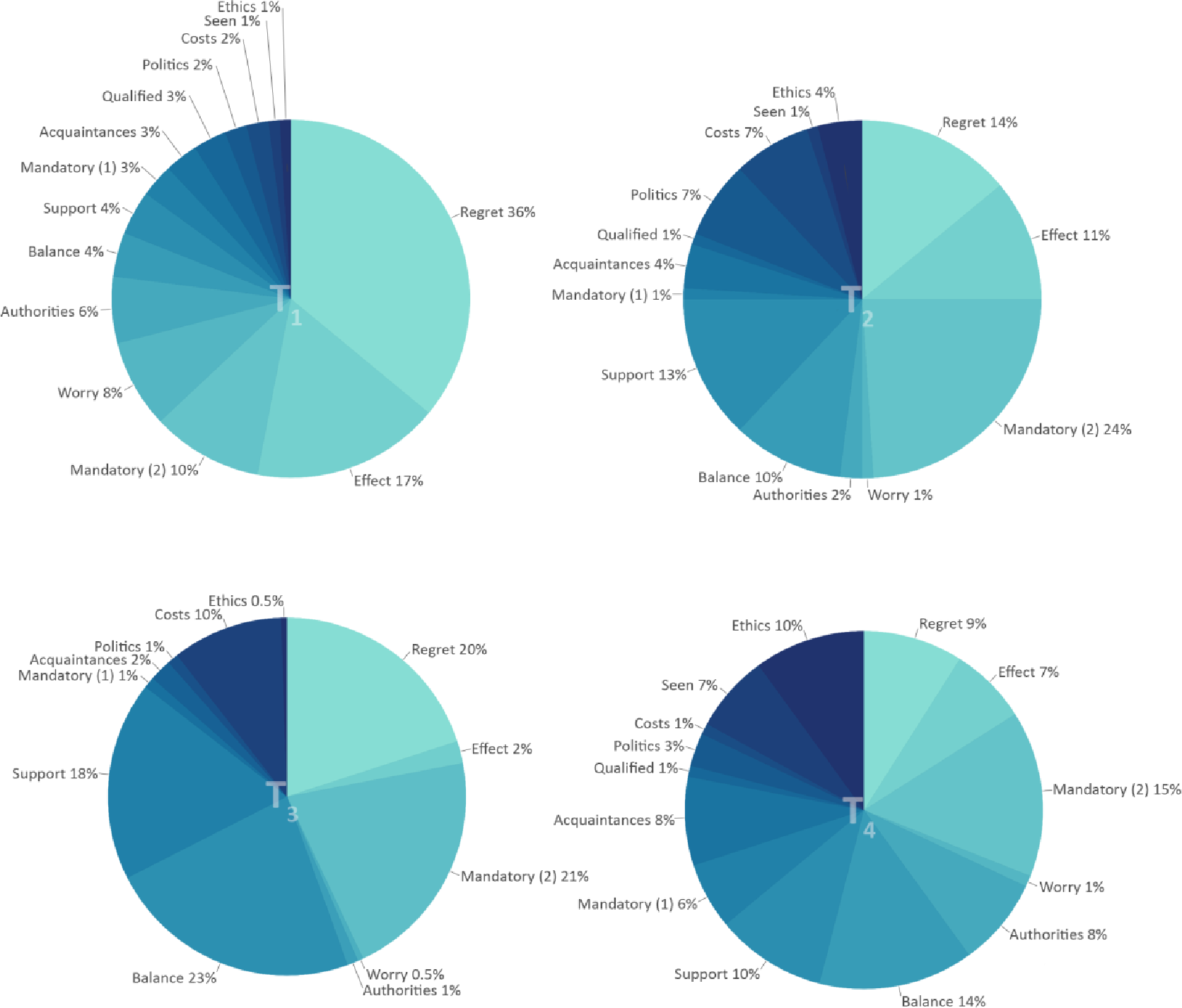

Supplement: S1 Fig — Note: Relative importance calculated in dominance analysis. Upper left: T1 = inquiry time point 1 (recruitment) R2 = 27.7, Upper right: T2 = inquiry time point 2 (after video information) R2 = 59.7, Lower left: T3 = inquiry time point 3 (after deliberation) R2 = 52.4, Lower right: T4 = inquiry time point 4 (one month after the Citizens’ Assembly) R2 = 51.5. (TIFF) [file pone.0317263.s001.tiff]

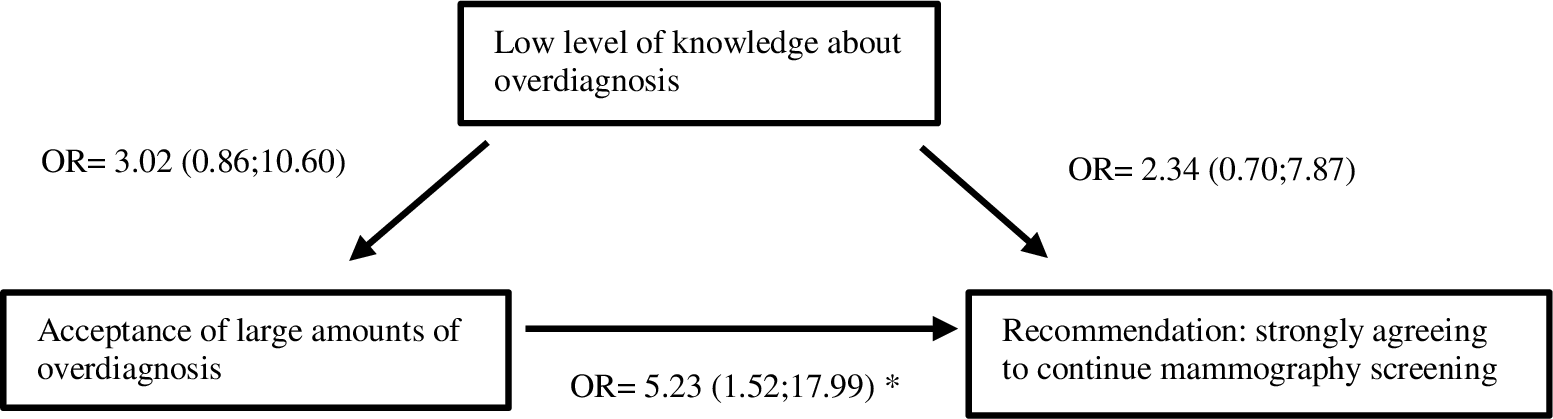

Supplement: S2 Fig — Note: Odds Ratio = OR (95% CI). Low level of knowledge about overdiagnosis was defined as answering zero one or two out of three knowledge questions correctly as opposed to answering all three questions correctly (high level of knowledge). Acceptance of much overdiagnosis was defined as accepting 100, 150 or 500 overdiagnosis per 1000 women invited for screening in 20 years as opposed to accepting low level of overdiagnosis (zero, one or five overdiagnosis per 1000 women invited over 20 years). (TIFF) [file pone.0317263.s002.tiff]

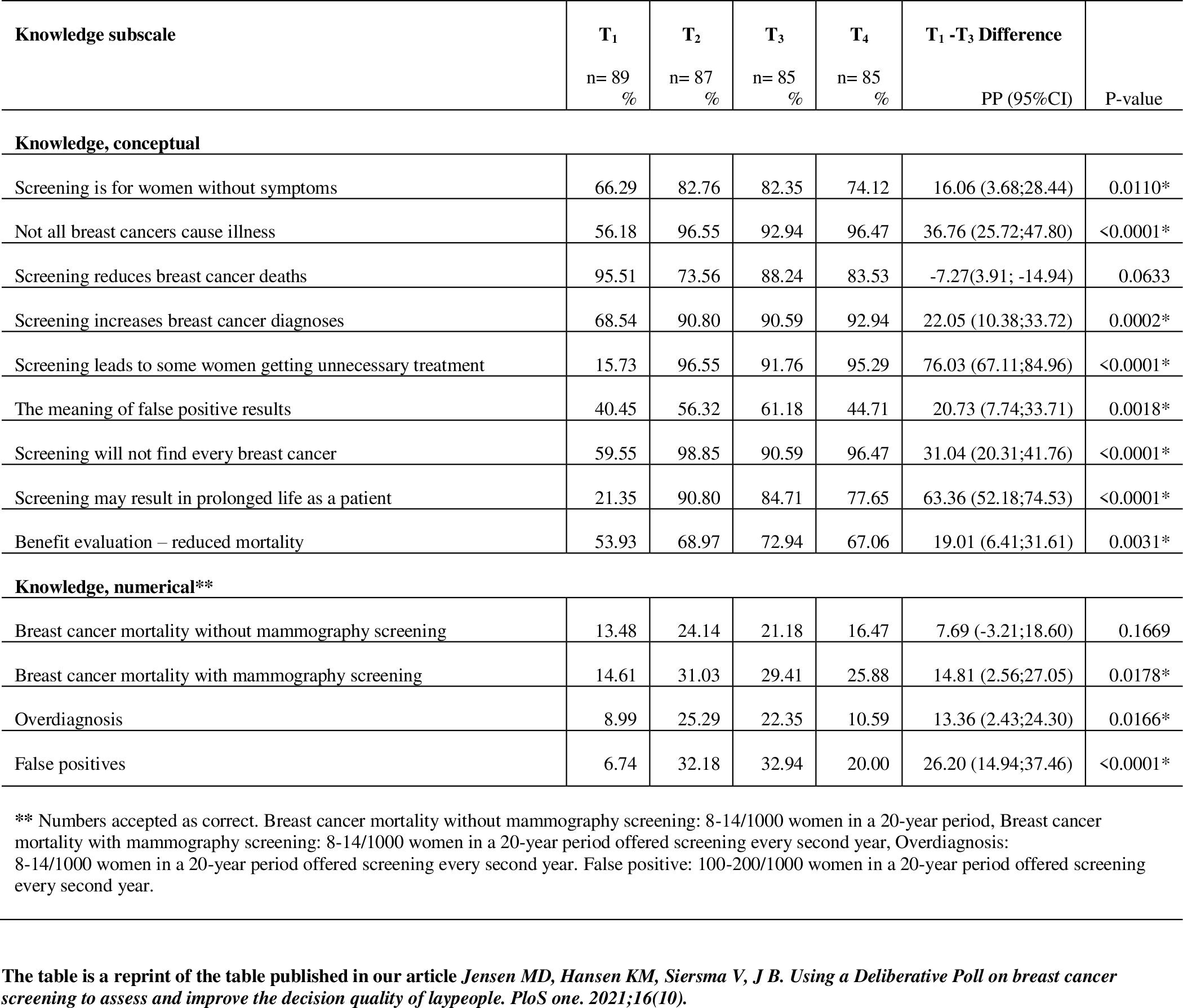

Supplement: S1 Table — Note: The table shows the level of knowledge at the four poll inquiry time points expressed as % correct answers to the 13 knowledge items. T1 = inquiry time point 1 (recruitment), T2 = inquiry time point 2 (after video information), T3 = inquiry time point 3 (after deliberation), T4 = inquiry time point 4 (one month after the citizens’ assembly). (TIFF) [file pone.0317263.s003.tiff]

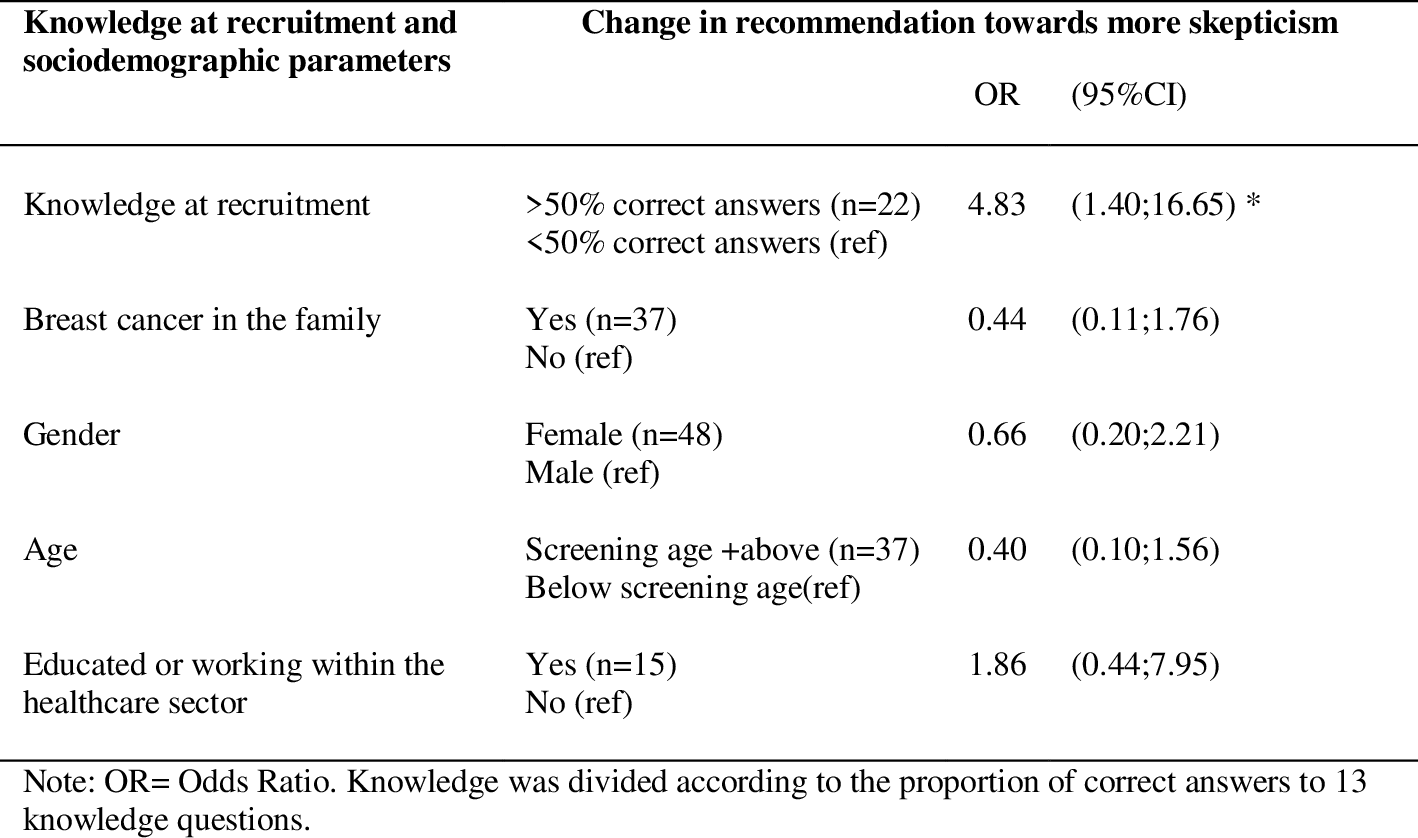

Supplement: S2 Table — Note: OR = Odds Ratio. Knowledge was divided according to the proportion of correct answers to 13 knowledge questions. (TIFF) [file pone.0317263.s004.tiff]

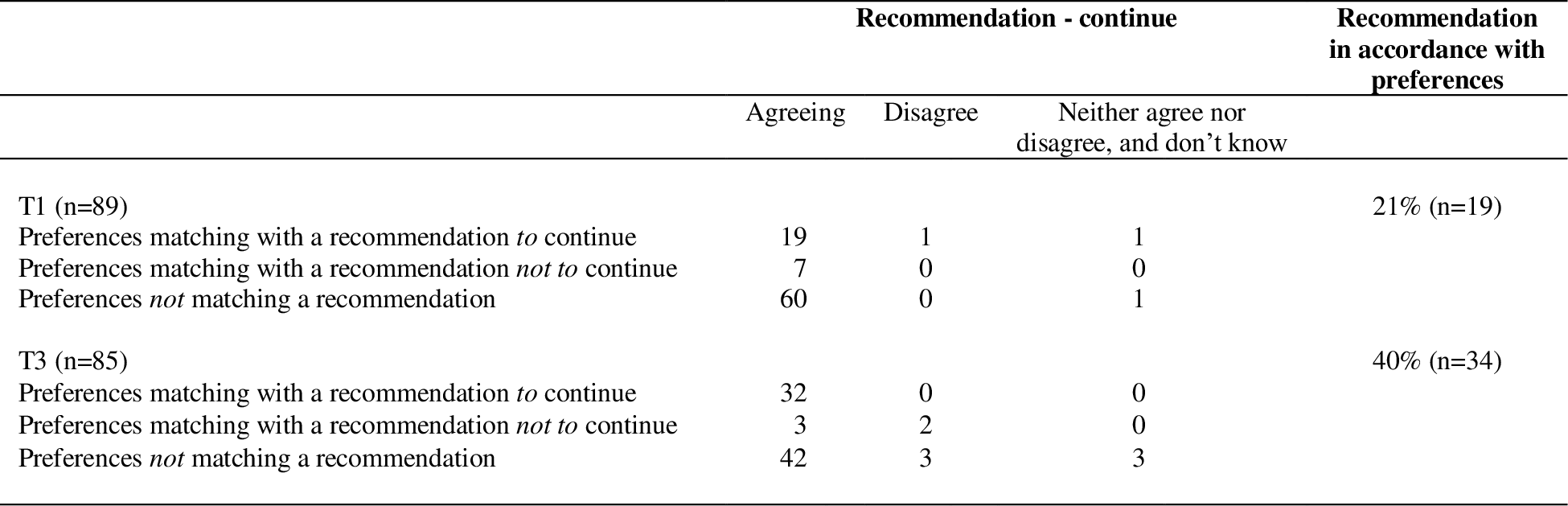

Supplement: S3 Table — Note: The table combines the answers to three questions. 1. Participants recommendation (agree or disagree to continue mammography screening in Denmark). 2. What participants consider to be an acceptable rate of mortality reduction in such a programme. 3. What participants consider to be an acceptable level of overdiagnosis in the programme. A preference matching with a recommendation to continue was defined as acceptance of one, two or five avoided deaths per 1000 women invited for mammography screening for 20 years + acceptance of 20, 100, 150 or 500 overdiagnoses per 1000 women invited in 20 years. A preference matching with a recommendation not to continue was defined as acceptance of 20 or 50 avoided deaths per 1000 women invited for mammography screening for 20 years + acceptance of zero, one or five overdiagnoses per 1000 women invited over 20 years. (TIFF) [file pone.0317263.s005.tiff]
