# Supplementary material for: Mammography screening: Eliciting the voices of informed citizens
Source: PLoS One. 2025 Jan 9;20(1):e0317263. doi: 10.1371/journal.pone.0317263 (PMC11717236; doi:10.1371/journal.pone.0317263)
Supplement: Attachment — Submitted filename: Response to Reviewers.docx [file pone.0317263.s006.docx]

**Response to Reviewers**

Journal Requirements:

1. Please ensure that your manuscript meets PLOS ONE's style requirements, including those for file naming. The PLOS ONE style templates can be found at

<https://journals.plos.org/plosone/s/file?id=wjVg/PLOSOne_formatting_sample_main_body.pdf> and

[https://journals.plos.org/plosone/s/file?id=ba62/PLOSOne_formatting_sample_title_authors_affiliations.pdf](https://eur02.safelinks.protection.outlook.com/?url=https%3A%2F%2Fjournals.plos.org%2Fplosone%2Fs%2Ffile%3Fid%3Dba62%2FPLOSOne_formatting_sample_title_authors_affiliations.pdf&data=05%7C01%7Cmadj%40sund.ku.dk%7C7459b93c8f164649f2ea08db10674337%7Ca3927f91cda14696af898c9f1ceffa91%7C0%7C0%7C638121808054387897%7CUnknown%7CTWFpbGZsb3d8eyJWIjoiMC4wLjAwMDAiLCJQIjoiV2luMzIiLCJBTiI6Ik1haWwiLCJXVCI6Mn0%3D%7C3000%7C%7C%7C&sdata=cx8b4X0DTkaHc%2B4g26JR2ePgc8p54iplhup89LkYE%2FU%3D&reserved=0)

*In the revised manuscript we have moved the quote by Joubert to page two to make it clear that the quote is not part of the title. To the best of our knowledge the manuscript now meets PLOS ONE's style requirements.*

*In the revised manuscript we have deleted the text “insert Fig.1.” and “insert Fig.2.”. In order to help with the layout we have uploaded a separate file called “Layout Fig1.and Fig2.” that shows how Fig1. and Fig2 can be formatted in relation to the text in the figure (heading and note).*

2. Please provide additional details regarding participant consent. In the ethics statement in the Methods and online submission information, please ensure that you have specified what type you obtained (for instance, written or verbal, and if verbal, how it was documented and witnessed). If your study included minors, state whether you obtained consent from parents or guardians. If the need for consent was waived by the ethics committee, please include this information.

*Information is now added about informed consent in the ethics statement in the methods section and online. Our study did not include minors.*

**Review Comments to the Author**

**Reviewer #1:**

The paper addresses an important topic, public engagement in decision-making about mammography screening. The intervention study is elaborate and yields relevant results about the preferences of informed laypeople regarding mortality reduction and overdiagnosis, the support for the continuation of mammography screening program in Denmark and the changes in knowledge after a deliberative polling intervention. However, in my view the paper needs some revisions regarding the presentation of the study rationale, method and some results:

1) One major point is that the intervention study has already been published in 2021 in previous paper in PLOS One (<https://doi.org/10.1371/journal.pone.0258869> ). The results in the present paper were not previously published, and also all figures and tables are original, this is all fine. However, the descriptions of the rationale of the study and most importantly of the intervention are too short in the present paper. E.g., the contents of the video and its duration are not described, the procedure of the deliberative polling day and the dependent measures are only very briefly summarized. The fact that the participants also received a fact sheet in addition to the video is only mentioned in the 2001 paper, not in the present paper. The authors refer to the 2001 paper several times in the methods section (p. 5ff.), however in my view also the present paper needs more details on the intervention and the measures to be interpretable on its own. As the results are being interpreted as effects of the intervention, the intervention and dependent variables need to be explained more precisely in the present paper.

*In the revised manuscript more information about the content of the video is added in the section ”The Deliberative Polling process”. In the same section we have also added that participants received a fact sheet as well as more information about the deliberative procedure and the dependent measures.*

2) The introduction section is relatively short and has some gaps; in my view, the following aspects need to be added:

a) a definition of deliberative polling (should be moved from p. 5 in the Method section to the Introduction).

b) a paragraph on prior research about how to communicate evidence about mammography screening,

c) a summary of prior research with regard to hypotheses 2 and 3. Also, the introduction does not refer to the study of the authors from 2021. Some relevant results of the study are currently presented in the methods section (p. 7) and should be moved to the introduction.

*In the revised manuscript we have moved the description of a Deliberative Poll from the methods section to the introduction. In addition, we elaborate more on the Deliberative Poll and the rationale of this method in the methods section.*

*In the revised manuscript we have included a paragraph on prior research about how to communicate evidence about mammography screening (Introduction section).*

*In the revised manuscript we describe and refer to our study from 2021 in the introduction section. In addition, the results described in the methods section is now moved to the introduction.*

3) Results I: Effect sizes should be described for all statistical tests, which is currently not the case.

*For analysis besides the chi-squared tests we present correlation coefficients and OR’s. In the revised manuscript we have added percentage point difference to the chi-squared/t-tests.*

4) Results II: Table 2 was partly unclear to me and should be clarified. E.g., do the correlation coefficients for the opinion items reflect correlations with change in recommendation or with change in knowledge (see title of table 2)? Also, I believe that the analysis of relative importance needs its own table or at least additional information; the percentage of relative importance with regard to the determination coefficients cannot be fully interpreted without showing the determination coefficients (and more information about these multiple regressions should be made available in the supplemental materials).

*In the revised manuscript we have specified in the header for the correlation coefficient (Table 2) that this is the coefficient for change in recommendation and change in knowledge score/item, and change in recommendation and change in opinion item, respectively. Similarly, for the headers for relative importance we have specified that these are the relative importance of the various changes in knowledge items for the change in recommendation, and the relative importance of the various changes in opinion items for the change in recommendation respectively.*

*It is not possible to give the RI analysis their own table as the calculation of RI entail many regression analysis (2 to the power #factorrs =8192 for the 13 knowledge items.) Instead we have added the R-squared of the full model in Table 2 (see table note) as well as S1_Fig. (see table note).*

5) Discussion I: Effect sizes should be taken into account when discussing the effects.

*In the revised manuscript we have included effect sizes in the discussion section.*

6) Discussion II: The discussion focuses on the unexpected result for H2. Some of the alternative explanations might be plausible for H2, but seem less plausible given that H1 was confirmed at the same time (e.g. if participants used gut-feelings instead of reflecting on knowledge (p. 12, line 23), why did still many of them change their recommendation?). Thus, I would recommend to reflect more on the combination of the results and also to elaborate a bit more on the results of the other hypotheses. The discussion of H2 could also be shortened a bit by moving some of the new results presented here to the results section.

*In the revised manuscript we have shortened the discussion of H2 a bit. Reflecting on H1 and H2 in combination, we agree with the reviewer and in the revised manuscript we have included a comment in relation to the reviewers point; finding it less likely that participants used gut feelings when many changed their recommendation.*

7) Discussion III: The section about limitations should be extended by the following points: What are the implications of a) the lack of a control group and b) the decision to use a representative sample of men and women, but not a representative sample of women only? Most previous studies studied women’s perceptions of mammography screening.

*In the revised manuscript (Strength and limitations section) we have included a discussion about the lack of a control group and our decision to use a representative sample (men and women) and not only woman.*

8) Conclusion: The conclusion currently reflects only parts of the main findings of the study with regard to the three hypotheses. Lines 11/12 are not clear to me – why does the balance between benefits and harms seem less important to the recommendations of informed lay people? The results of the study pointed into another direction.

*In the revised manuscript our conclusions regarding all three hypotheses are described. In addition, we have reframed the bit about the balance between benefits and harms.*

Minor points:

9) Hypothesis 2 is sometimes about knowledge gain (p. 4, 12), sometimes about change in knowledge (p. 9, Table 2), which is not necessarily equivalent. It would be helpful for readers if one of the two expressions would be used consistently when discussing H2.

*In the revised manuscript we use only “change in knowledge”.*

10) Descriptive data about the changes in knowledge, as well as absolute levels of knowledge, is currently missing and could be added as a table in the supplementary materials. This could also be helpful for interpreting the results of H2.

*In the revised manuscript we describe in the introduction section that participants’ knowledge about mammography screening increased markedly as a result of the Deliberative Polling process “at recruitment only 1% of participants were able to answer two-thirds of the knowledge questions correctly. After video information the proportion was 56% (With reference to our previous paper).*

*In the revised manuscript we have also included the supplementary table “Level of knowledge”. NB: this is a reprint of the table we published in our previous article* *Jensen MD, Hansen KM, Siersma V, J B. Using a Deliberative Poll on breast cancer screening to assess and improve the decision quality of laypeople. PloS one. 2021;16(10). We write in the table note that this is a reprint.* ***Is it okay to publish a reprint like this?***

11) Conclusion: The framing of the result of H3 is interesting: “Participants’ recommendations about the continuation of mammography screening did not, to a large extent, reflect their preferences regarding mortality reduction and overdiagnosis in the programme.” (p. 16) vs. “In line with our third hypothesis, recommendations were more in line with preferences regarding mortality reduction and overdiagnosis after information and deliberation compared with before.” (p. 12). Of course, both statements are correct. But which is the main point that should be included in the abstract and in the conclusions? Personally, I would prefer that both aspects are presented equitably.

*In the revised manuscript both aspects are presented in the abstract as well as the conclusion.*

12) P. 3, 22-24: Some more current studies should be added.

*In the revised manuscript we have added the following studies:*

*Yu J, Nagler RH, Fowler EF, Kerlikowske K, Gollust SE. Women’s Awareness and Perceived Importance of the Harms and Benefits of Mammography Screening: Results From a 2016 National Survey. JAMA internal medicine. 2017;177(9):1381-2.*

*Abelson J, Tripp L, Brouwers MC, Pond G, Sussman J. Uncertain times: A survey of Canadian women's perspectives toward mammography screening. Preventive Medicine. 2018;112:209-15.*

13) References [23] and [34] are very general and should be replaced with more specific references that are a bit closer to the present context of medical decision-making.

*In the revised manuscript we have deleted the following “general” bit and the referance: “This also applies to the public’s decision-making in a more general sense (Ref. 23: Tversky A, Kahneman D. The framing of decisions and the psychology of choice. Science. 1981;211(4481):453-8.)*

14) A reference is missing for the theory of cognitive dissonance (p. 13, line 3)

*In the revised manuscript the reference is added.*

15) S1_Fig.: I would recommend to use bar charts instead of pie charts.

*Because the relative importance adds up to 100% we find that pie charts to be natural.*

16) S2_Table: Absolute frequencies should be added (e.g., number of participants educated or working in the healthcare sector).

*In the revised manuscript absolute frequencies are added to the table.*

**Reviewer #2:**

17) Very interesting findings and a complex intervention!
One question: On page 9 you write: "Learning (change in knowledge index) was not correlated to a change in recommendation, correlation 0.03 (95%CI -0.19-12 0.24) (Table 2). On the contrary, a change in opinion towards viewing the harms of screening carrying more weight than the benefits, was correlated to a change in recommendation towards being more skeptical learning was not correlated to a change in recommendation." In our experience (not research!) this is not an contradiction as information (e.g. statistical facts) has very different meanings for individuals. That is how decisions aids work (give information + als "what does this mean fo me?)
Maybe ist is possible to summarise in short (e.g. in the abstract) what made people change their recommendation (if it is not knowlege and if the opinion items had relatively little importance).

*Thank you for the comment. Unfortunately, we can only hypothesize on this based on our results and study design and therefore we only discuss this in our discussion section. We agree that it could be interesting to do qualitative work in relation to a Deliberative Poll which could probably shed more light on what made people change their recommendation.*

**Reviewer #3:**

Thank you for the opportunity to review the paper „Mammography screening: eliciting the voices of informed citizens“
Aim of the present study was to examine the public’s recommendations about the continuation of mammography screening and the preferences regarding acceptable rates of mortality reduction and overdiagnosis.

18) For the public involvement, an online Deliberative Poll was held in Denmark in 2020 and outcoemes were assessed on four time points. Results on knowledge and opinion items have already been published in PLoS One in 2021. This publication is referenced in the present manuscript (reference 31) in order to refer to the methodological approach as well as previous results. However, these references do not make it clear that the present manuscript is a secondary analysis, or at least a further analysis of the data already published. If it seems appropriate to submit more than one publication for a study, the authors should describe and justify this procedure transparently.

*Thank you for the good comment.* *In the revised version of the manuscript we have included information about our previous study in the introduction section (the aim and main results) just before the aim of the present study. We hope this contribute to transparency. The aim in the present paper is very different from the aim in the first paper and the results in the present paper is not previously published.*

Apart from that, the subject is important and the method for public involvement is innovative. The results are relevant and could form the basis for further research into the relationship between knowledge, attitudes and recommendations for screening. Knowledge on these associations (or the lack of them) could also be important for interventions to enhance individual informed decisions.
In the following, find some smaller comments on the manuscript and supplementing materials:

19) Page 15, lines 12 ff: The attribution of the descriptions in the text to the items and results in Table 2 is not entirely clear. Possibly the effects could be represented here, as in line 11.

*In the revised manuscript we have added the following text “(S1 Table, after information and deliberation 40% made a recommendation that was in accordance with their preferences regarding mortality reduction and overdiagnosis”) in order to link the text about 60% (100-40%) to the S1 Table.*

20) Table S2 is introduced only in the discussion. This is rather unusual, and it would be better to take up only results that have already been presented in advance in the discussion.

*In the revised manuscript results shown in the S2 Table are presented in the result section as well.*

21) The arrows and numbers in Fig. 1 should be explained in the legend.

*In the revised manuscript we explain in the note (Fig.1) that the arrows with numbers indicate specific changes in recommendation between time points.*

22) Fig. 2: To avoid framing of data, the axes should always go up to 100 in the representation of percent.

*In the revised manuscript the axes in Fig.2 go up to 100.*

23) Fig. S1: In the current display, the assignment of the graphics to the times is missing.

*It is actually written in the center of each figure (text written with opacity). In the revised manuscript we have added this information in the figure note.*
