# Supplementary material for: Mammography screening: Eliciting the voices of informed citizens
Source: PLoS One. 2025 Jan 9;20(1):e0317263. doi: 10.1371/journal.pone.0317263 (PMC11717236; doi:10.1371/journal.pone.0317263)
Supplement: Attachment — Submitted filename: Response to Reviewers.docx [file pone.0317263.s007.docx]

PONE-D-22-34061R1

Mammography screening: eliciting the voices of informed citizens

PLOS ONE

Dear Dr. Jensen,

Thank you for submitting your manuscript to PLOS ONE. After careful consideration, we feel that it has merit but does not fully meet PLOS ONE’s publication criteria as it currently stands. Therefore, we invite you to submit a revised version of the manuscript that addresses the points raised during the review process.

==============================

In the light of earlier revising and addressing the points of the reviewers, it may be another effort to process the comments below. But major concerns need to be addressed for continuing with the manuscript. Some of them may be solved by just better explaining the details or the thoughts behind.

==============================

Please submit your revised manuscript by Dec 01 2023 11:59PM. If you will need more time than this to complete your revisions, please reply to this message or contact the journal office at [plosone@plos.org](mailto:plosone@plos.org). When you're ready to submit your revision, log on to <https://www.editorialmanager.com/pone/> and select the 'Submissions Needing Revision' folder to locate your manuscript file.

Please include the following items when submitting your revised manuscript:

- A rebuttal letter that responds to each point raised by the academic editor and reviewer(s). You should upload this letter as a separate file labeled 'Response to Reviewers'.
- A marked-up copy of your manuscript that highlights changes made to the original version. You should upload this as a separate file labeled 'Revised Manuscript with Track Changes'.
- An unmarked version of your revised paper without tracked changes. You should upload this as a separate file labeled 'Manuscript'.

If you would like to make changes to your financial disclosure, please include your updated statement in your cover letter. Guidelines for resubmitting your figure files are available below the reviewer comments at the end of this letter.

If applicable, we recommend that you deposit your laboratory protocols in [protocols.io](http://protocols.io) to enhance the reproducibility of your results. [Protocols.io](http://Protocols.io) assigns your protocol its own identifier (DOI) so that it can be cited independently in the future. For instructions see: [https://journals.plos.org/plosone/s/submission-guidelines#loc-laboratory-protocols](https://eur02.safelinks.protection.outlook.com/?url=https%3A%2F%2Fjournals.plos.org%2Fplosone%2Fs%2Fsubmission-guidelines%23loc-laboratory-protocols&data=05%7C01%7Cmadj%40sund.ku.dk%7Cf279e3cbe12d453e184208dbcf560234%7Ca3927f91cda14696af898c9f1ceffa91%7C0%7C0%7C638331740540605517%7CUnknown%7CTWFpbGZsb3d8eyJWIjoiMC4wLjAwMDAiLCJQIjoiV2luMzIiLCJBTiI6Ik1haWwiLCJXVCI6Mn0%3D%7C3000%7C%7C%7C&sdata=Dspt0eBcsqIe9XB4kd3G7vDz1l%2BLCyBXmoirZgoCI0Y%3D&reserved=0). Additionally, PLOS ONE offers an option for publishing peer-reviewed Lab Protocol articles, which describe protocols hosted on [protocols.io](http://protocols.io). Read more information on sharing protocols at [https://plos.org/protocols?utm_medium=editorial-email&utm_source=authorletters&utm_campaign=protocols](https://eur02.safelinks.protection.outlook.com/?url=https%3A%2F%2Fplos.org%2Fprotocols%3Futm_medium%3Deditorial-email%26utm_source%3Dauthorletters%26utm_campaign%3Dprotocols&data=05%7C01%7Cmadj%40sund.ku.dk%7Cf279e3cbe12d453e184208dbcf560234%7Ca3927f91cda14696af898c9f1ceffa91%7C0%7C0%7C638331740540605517%7CUnknown%7CTWFpbGZsb3d8eyJWIjoiMC4wLjAwMDAiLCJQIjoiV2luMzIiLCJBTiI6Ik1haWwiLCJXVCI6Mn0%3D%7C3000%7C%7C%7C&sdata=Y5GHvkQgqNGve1omkyw6ivl3NsGsE3AXzBU%2Fvvfo534%3D&reserved=0).

We look forward to receiving your revised manuscript.

Kind regards,

Felix G. Rebitschek
Academic Editor
PLOS ONE

**Additional Editor Comments:**

**Majors**

• What is the research question?
o Do the authors want to inform about potential advantages and effects by the specific method Deliberative Poll? Is it a paper about a method? Then, more justification is needed about why selecting the mammography case?
o Do the authors want to inform an issue in breast cancer guideline development, because the study reveals neglected attitudes of informed citizens that from now on can be used? Is it a paper about qualitative insights of mammography beliefs and attitudes in a Western population?

*Thank you for the comment. The focus of our first paper from the project (“Using a Deliberative Poll on breast cancer screening to assess and improve the decision quality of laypeople”, published 2021 in PLOS ONE) was whether or not our experimental setup – the Deliberative Poll - approached the purpose or potential of this type of citizens’ involvement in decision making regarding continuing the Danish mammography screening program. Ie.a stronger focus on the method of Deliberative Polling on this complex issue.*

*The present study has a more substantial focus. I.e. how information and deliberation affected citizens’ recommendations about continuing the Danish mammography screening program. The primary aim was to determine an informed public’s recommendations about the continuation of the program in Denmark as well as to determine whether an informed recommendation was related to preferences regarding mortality reduction and overdiagnosis. The secondary aim was to examine how the process of information and deliberation affected opinions related to mammography screening. This is now highlight and explained in the introduction to the paper.*

• What is a public’s recommendation? What is the role of the citizens exactly? Why they give recommendations to whom – is there any real-world equivalent?

*Thank you for the comment. Why, when and how citizens should be involved in policy matters related to screening can be debated. Studies like ours cannot prove or disprove political theories about citizens’ involvement - whether or not we should involve citizens in decision-making about mammography screening. The focus of this paper is how information and deliberation affected citizens’ recommendations about continuing the program. I.e. descriptively. We hope this is clearer in the revised manuscript.*

*According to Degeling, Carter and Rychetnik in their paper “Which public and why deliberate? – A scoping review of public deliberation in public health and health policy research” the public is often constructed in one of three ways when they are engaged in healthcare matters; either as advocates, consumers or as citizens (the “pure” public). In the revised manuscript we reference this paper and give some examples of public involvement in Danish healthcare matters.*

o What is the exact subject of the study – an age-stratified population-based mammography screening without risk assessment? Is it an offer, obligatory, biannualy, yearly, age-range? This clearly affects the interpretation of the study – I could read it off from figure/table captions, but this is too central.

*Thank you for the comment. In the revised manuscript we describe the Danish programme (the subject of the study) in the beginning of the introduction.*

• What is the argument for applying the method? This needs much more argumentation
o “[other methods’] limited representativeness of the wider population” – with 100 people the authors do not reach population representativeness, so what is meant by that (please define in which way the author’s sample is representative)? The generalisation of a convenience sample is negligible if it is about attitudes. Is the point about a stratified sampling to assure that certain vulnerable subgroups are included? How do the authors show the “representativeness” advantage?

*The sample is never representative or not. It is always a matter of degree, but in our case, it is quite good. Both in terms on socio demographics (e.g., gender, age, education and marital status) and history of breast cancer our sample is not statically different from the population (see table 1). The sampling is drawn from a random selected panel (e.g., is not self-selected). Participants were incentivized to participate, and much effort was put into the recruitment. The recruitment and the participants’ characteristics is described in detailed in our method-paper published in PloS ONE in 2021* [*https://www.ncbi.nlm.nih.gov/pmc/articles/PMC8530304/pdf/pone.0258869.pdf*](https://www.ncbi.nlm.nih.gov/pmc/articles/PMC8530304/pdf/pone.0258869.pdf)*. We now include some information on this and provide a direct reference to this paper.*

The “range of arguments” is also unclear. There is some plausibility that the authors reveal more aspects when they do qualitative research with 100 instead of 10 participants, but why not 20 or 30? What makes this method reveal sufficiently broader arguments and do the authors have evidence for that (references)? Particularly in the light of so much interesting qualitative research with 10-15 participants… Later I found out that you do not provide qualitative insights, so we are talking about a cohort survey study.

*We agree that there is much interesting qualitative research with 10-15 participants including the qualitative studies based on Citizens’ Juries about screening referenced in our manuscript. We did a survey study and in the revised manuscript the abstract now mentions “survey data” to highlight this in the beginning. Our aim of 100 participants was based on various power tests in term if the group of participants would be a large enough group to find relevant statistical differences.*

• The third hypothesis requires more background, because the authors want to compare two flying bullets: a changing recommendation-related judgment (an expression of preference) and an evaluation of a required minimum benefit and maximum harm. The authors here touch the fields of preference elicitation and shared decision-making and should provide more conceptual ground on the key-strength of the described study: linking option knowledge with underlying preferences and final intentions/recommendations. What was done in the supplementary figure on overdiagnoses should have be done on the full knowledge, the most aggregated preference assessment that the authors have, with recommendation.

*Thank you for the comment. In the revised manuscript (introduction section) we elaborate on how group deliberation is believed to affect inconsistent opinions which function as background for our third hypothesis.* *In the revised manuscript we refer to this information in the description of our third hypothesis*

• Method section:
o with regard to your representativeness argument, information on recruitment needs to be extended here: sampling, frame, inclusion/exclusion criteria, participation rates, risk of a topical bias – how were candidates approached? Avoid the bold statements (p.7,l12-19; not only because of the age range 18-70 and the online study requirements, but also because in crossed cells the impressive alignment with the general Danish population (on the main factors) does not hold; even for crossed cells of age x education x gender 100 people are not sufficient).

*Thank you for the comment. In the revised manuscript the recruitment process is described in more detail and a flow diagram for the recruitment is now added (Fig.1). The flow diagram includes the number of participants approached as well as excluded. There is also a reference to the method-paper mention above.*

o please restructure into distinct paragraphs for study material and procedure

*Thank you for the comment. In the revised manuscript we have restructured the methods section.*

o why the separate analyses for the knowledge on overdiagnosis and mortality reduction, but not on the other eleven items?

*Thank you for the comment. We made a separate analysis concerning what we refer to as participants “preferences” regarding overdiagnosis and mortality reduction (Fig.4) and a separate analysis regarding the relations between knowledge about overdiagnosis, acceptance of overdiagnosis and recommendation after information and deliberation (T_3_) (S2.Fig). Mortality reduction and overdiagnosis are often highlighted as the most important benefit and harm of the program and we therefore found it interesting to look closer at these two aspects.*

o Provide more information about the used measures here in a separate measures paragraph (for instance the preference assessments, such as acceptable overdiagnosis.

*Many of the survey questions were developed specifically for this project. We follow general survey methodology and try to minimize the cognitive demand on participants by applying classic Likert scales when every possible. Other items were developed and tested in previous Deliberative Polls (mostly the question on evaluation the deliberative and the briefing materials). The questionnaires were tested in a focus group before the distribution, and we also reviewed input from the Survey company who collected the data (Gallup).*

o Please provide the full items including false options for knowledge (beyond the supplementary reprint)
--> What is the reference for the ground truth and for the range that you scored correct in knowledge, numerical?
--> What is the reliability of this assessment?

*Thank you for the comment. The full knowledge items are now presented in Fig.2.*

*The information presented in the video and fact sheet functioned as “ground truth”. In the method section “Video information” we have references to the Cochrane report, The Danish Quality Database of Mammography Screening (Annual Report 2019), Statistics Denmark and an economic evaluation of the Danish programme. They were used as sources for mortality reduction, overdiagnosis, etc. presented in the video. In the revised manuscript we have given the information about the video in its section and the section “Data collection and trial outcome” we have added that the video information and fact sheet functioned as “ground truth” concerning knowledge.*

*We do not have a reference for the range we accepted. We choose to accept a range of numbers as correct because we acknowledge the uncertainty surrounding the numbers. See note in Table 1. for the range accepted as correct. We mention the uncertainty at the beginning of the video to be transparent about this.*

*We could have made a test-retest (for example with 2-4 weeks span) in order to test the reliability. However, we know from psychometric research that items that are unambiguous, easy to understand and have high relevance have higher reliability compared to items with the opposite characteristics. In our study we aimed for high content validity and thereby indirect we tried to increase reliability.*

• Results
o Proportions of recommendation statements should be accompanied by information about uncertainty around those estimates.

*Our Fig.3 displays participants’ responses to the question “To what extent do you agree that we should continue mammography screening in Denmark” Responses (strongly agree, agree etc.) are shown in %. In the revised manuscript we have included 95% CI.*

o Fig. 2:
--> I do not understand the mortality-reduction item and I doubt that laypeople could so. Is this a problem of an English translation?

*Thank you for the question. We did three think-aloud tests (in Danish) to get an understanding of how lay individuals who had not viewed the mammography screening video understood our questions and we modified the wording accordingly. This information is added to the revised manuscript.*

*We have adjusted the translation slightly, so it is truer to the actual question asked. Please also remember that the participants answer the question after a longer discussion on the issue.*

--> Have you binned the responses or was it multiple choice?

*Thank you for the comment. It was a multiple choice. In the revised manuscript this information is added to the figure text.*

--> The formatting needs to be checked against PLOSONE standards

*Thank you for the comment. The manuscript no longer contains multiple columns concerning figure text.*

--> Please insert Confidence intervals or any other indication of uncertainty around the survey estimates.

*The bar chart shows the percentage of participants accepting different thresholds for mortality reduction and different levels of overdiagnosis. In the revised manuscript we have included 95% CI.*
o Table 1.
--> Is it [%] in each column?

*Thank you for the comment. Yes. In the revised manuscript, we have added “%” to each column (beside % in the header).*

--> A dominance analysis with such a small sample over so many items requires information about what is the uncertainty around the estimate of relative importance for each item
*While naïve uncertainty information, e.g. 95% confidence intervals on the estimates of relative importance, may be obtained by bootstrap, this will require quite some time investment, primarily to execute these calculations. Furthermore, there will be considerable trouble representing such uncertainty information in the figures in an understandable way, and trouble on how to interpret this information relative to pointing out the most important factors. We view inclusion of uncertainty estimates in the dominance analysis an (time-consuming) exercise that will cause much confusion and not lead to different conclusions.*

• Discussion:
o limitations by your method needs to more elaboration, starting with the generalisability of your findings to which population. *Thank you for the comment. In the revised manuscript we include information about the lack of cross-cell representativeness as well as volunteer bias.*

o what is role of modality for deliberate polls – offline, hybrid, online?

*Thank you for the comment. In the revised manuscript we mention the implications of conducting the citizens assembly online instead of offline as originally planned.*

o please elaborate on the methodological point of equal considerations of input by women among your participants (who are or will be offered mammography screening) and other participants (e.g., men) in determining a public’s consideration – within participatory research literature distinguishing those who are directly affected by a subject and those who are not.

*Thank you for the comment. In the revised manuscript we elaborate on our choice to include men as well as women (people who are directly affected and those who are not).*

• Please rewrite the abstract which cannot be understood by a reader without the authors‘ background (what is a recommendation by laypeople for whom, type of mammography screening, what is the method, representativeness in which way, result focus on the method’s contribution).

*Thank you for the comment. We have rewritten the abstract.*

• Language editing, please let a native lecturer go over the manuscript which contains many types of errors (e.g., p.3, l.14 incomprehensible; which evaluation?; p.4,l.22) and unusual expressions.

*Thank you for the comment. The revised manuscript has been edited.*


**Minors**
• Please provide the correct permanent video link.

*The video contains the authors' names and the link is therefore blinded (only the editor is provided with the permanent link so far). In the final manuscript, the real link will be presented.*

• Why is public engagement in decision-making a way to address different preferences of experts?

*In the revised manuscript we have reframed the sentence and instead of “address” we write “supplement”.*

• What is opinion stability and opinion consistency – difference? References? Do we talk about attitudes?

*In our manuscript, we only mention “opinion stability” and “opinion consistency” when we explain the focus of our previous study. An in-depth description of these measures can be found there. In the revised manuscript we have added how we operationalised stability as correlations within opinion items at different time points and consistency as correlations between the 14 opinion items within each time point.*

• Please check for consistent capitalization (different methods)?

*Thank you for the comment.*

• How could the people from the survey assemble physically – or was it fully online?

*It was fully online. In the revised manuscript we write “online” more often.*

• What is “Citizen’s Juries”?

*In the revised manuscript we describe the Citizen Jury method in the introduction section.*

• How did you select “neutral” moderators?.

# *Thank you for the comment. We have reframed the sentence in the revised manuscript. The moderators were primarily school teachers without special knowledge about mammography screening. They were instructed (in writing as well as at the onboarding meeting prior to the assembly) to take a neutral position. At the end of the citizens assembly participants evaluated the moderators. 74% somewhat or strongly disagreed with the statement “The moderators in my group aimed at pushing my opinion in a certain direction” (Se our first article, Using a Deliberative Poll on breast cancer screening to assess and improve the decision quality of laypeople,* [*S3 Table.*](https://journals.plos.org/plosone/article/file?type=supplementary&id=10.1371/journal.pone.0258869.s007) *)*

• Please move derived hypothesis (p.9.l17-20) up to the introduction

*Unfortunately, we do not understand this comment. P9, l17-20 is part of the paragraph “statistical analysis” and it says: “Likewise, the relative importance of the opinion items to the recommendation at each of the four inquiry time points was depicted in pie charts to determine which opinion carried most weight for the recommendation at each of the stages in the Deliberative Poll. A paired chi-squared test was used to test the change in acceptance of high levels of overdiagnosis as well as acceptance of a mortality reduction of one between T_1_ and T_3_.”*

• What does mean: “an informed recommendation is RELATED to preferences” ? informed decisions, for instance, are defined by an alignment of the benefit-harm evaluation of options, option knowledge and the chosen option (Theresa Marteau)

*Thank you for the comment. In the revised manuscript we define what we mean by “preferences regarding mortality reduction and overdiagnosis” at the end of the introduction section: “…an informed recommendation was in line with what participants consider to be an acceptable mortality reduction and an acceptable level of overdiagnosis in a mammography screening program (referred to as their preferences regarding mortality reduction and overdiagnosis)”*

• Direct references for “Mammography screening is controversial”.

*Thank you for the comment. We write “much debated” instead.*

• Check for an update of Mammography screening, e.g. Germany IQWIG recommended extension to new age groups

*Thank you for the comment. In the revised manuscript we have included that the EU Commission and the German IQWIG recommend extending the program.*

• Ref. for benefit overestimation worth to consider: Gigerenzer, G., Mata, J., & Frank, R. (2009). Public knowledge of benefits of breast and prostate cancer screening in Europe. Journal of the National Cancer Institute, 101(17), 1216-1220.

*Thank you for the comment. Besides the systematic review by Hoffmann et al. we reference Gigerenzer et. al in the revised manuscript.*

• Please provide a reference for P.6.,l. 20-22 – consider moving the sentence to the introduction of deliberate poll

*In the revised manuscript we have included a reference to the Office of Research and Innovation (ethics section).*

• P.10, l.1-3. The sentence is unclear to me.

*Page 10. Lines 1-3 in our document say: “The study succeeded in recruiting a sample that was statistically representative of the adult Danish population on sociodemographic parameters as well as on attitudes and knowledge about mammography screening (Table 1).” Do you mean another sentence?*

• Is correlation 0.03 => r = .03 (please check results descriptions against APA/PLOSONE)

*We have kept leading zeros in the manus.*

• Could you please restructure the supplementary table about recommendation-continuing: here I read 3 agree at t3 with “continue” although they fall under “preferences matching with a recommendation not to continue”…? Unclear.

*Thank you for the comment. You read it as we intended. In the revised manuscript we have added information to the table note. Now it says the following:*

*Note: The table combines the answers to three questions. 1. Participants' recommendations (agree or disagree to continue mammography screening in Denmark). 2. What do participants consider to be an acceptable rate of mortality reduction? 3. What do participants consider to be an acceptable level of overdiagnosis? A preference matching with a recommendation to continue was defined as acceptance of one, two or five avoided deaths per 1,000 women invited for mammography screening for 20 years + acceptance of 20, 100, 150 or 500 overdiagnoses per 1,000 women invited in 20 years. A preference matching with a recommendation not to continue was defined as acceptance of 20 or 50 avoided deaths per 1,000 women invited for mammography screening for 20 years + acceptance of zero, one or five overdiagnoses per 1,000 women invited over 20 years”. We hope the table makes sense now.*

[Note: HTML markup is below. Please do not edit.]

Reviewers' comments:

Reviewer's Responses to Questions

**Comments to the Author**

1. If the authors have adequately addressed your comments raised in a previous round of review and you feel that this manuscript is now acceptable for publication, you may indicate that here to bypass the “Comments to the Author” section, enter your conflict of interest statement in the “Confidential to Editor” section, and submit your "Accept" recommendation.

Reviewer #1: All comments have been addressed

2. Is the manuscript technically sound, and do the data support the conclusions?

The manuscript must describe a technically sound piece of scientific research with data that supports the conclusions. Experiments must have been conducted rigorously, with appropriate controls, replication, and sample sizes. The conclusions must be drawn appropriately based on the data presented.

Reviewer #1: (No Response)

3. Has the statistical analysis been performed appropriately and rigorously?

Reviewer #1: (No Response)

4. Have the authors made all data underlying the findings in their manuscript fully available?

The [PLOS Data policy](https://eur02.safelinks.protection.outlook.com/?url=http%3A%2F%2Fwww.plosone.org%2Fstatic%2Fpolicies.action%23sharing&data=05%7C01%7Cmadj%40sund.ku.dk%7Cf279e3cbe12d453e184208dbcf560234%7Ca3927f91cda14696af898c9f1ceffa91%7C0%7C0%7C638331740540605517%7CUnknown%7CTWFpbGZsb3d8eyJWIjoiMC4wLjAwMDAiLCJQIjoiV2luMzIiLCJBTiI6Ik1haWwiLCJXVCI6Mn0%3D%7C3000%7C%7C%7C&sdata=uetASWD5umxBT8WdZb7uxjnjXisybQbuoGkHHmEAX%2FI%3D&reserved=0) requires authors to make all data underlying the findings described in their manuscript fully available without restriction, with rare exception (please refer to the Data Availability Statement in the manuscript PDF file). The data should be provided as part of the manuscript or its supporting information, or deposited to a public repository. For example, in addition to summary statistics, the data points behind means, medians and variance measures should be available. If there are restrictions on publicly sharing data—e.g. participant privacy or use of data from a third party—those must be specified.

Reviewer #1: (No Response)

5. Is the manuscript presented in an intelligible fashion and written in standard English?

PLOS ONE does not copyedit accepted manuscripts, so the language in submitted articles must be clear, correct, and unambiguous. Any typographical or grammatical errors should be corrected at revision, so please note any specific errors here.

Reviewer #1: (No Response)

6. Review Comments to the Author

Please use the space provided to explain your answers to the questions above. You may also include additional comments for the author, including concerns about dual publication, research ethics, or publication ethics. (Please upload your review as an attachment if it exceeds 20,000 characters)

Reviewer #1: (No Response)

7. PLOS authors have the option to publish the peer review history of their article ([what does this mean?](https://eur02.safelinks.protection.outlook.com/?url=https%3A%2F%2Fjournals.plos.org%2Fplosone%2Fs%2Feditorial-and-peer-review-process%23loc-peer-review-history&data=05%7C01%7Cmadj%40sund.ku.dk%7Cf279e3cbe12d453e184208dbcf560234%7Ca3927f91cda14696af898c9f1ceffa91%7C0%7C0%7C638331740540605517%7CUnknown%7CTWFpbGZsb3d8eyJWIjoiMC4wLjAwMDAiLCJQIjoiV2luMzIiLCJBTiI6Ik1haWwiLCJXVCI6Mn0%3D%7C3000%7C%7C%7C&sdata=2zYs8ZdmUSXR%2B8jkMXo4YSgEYZK1bWwK4pQVOD534mM%3D&reserved=0)). If published, this will include your full peer review and any attached files.


If you choose “no”, your identity will remain anonymous but your review may still be made public.


**Do you want your identity to be public for this peer review?** For information about this choice, including consent withdrawal, please see our [Privacy Policy](https://eur02.safelinks.protection.outlook.com/?url=https%3A%2F%2Fwww.plos.org%2Fprivacy-policy&data=05%7C01%7Cmadj%40sund.ku.dk%7Cf279e3cbe12d453e184208dbcf560234%7Ca3927f91cda14696af898c9f1ceffa91%7C0%7C0%7C638331740540605517%7CUnknown%7CTWFpbGZsb3d8eyJWIjoiMC4wLjAwMDAiLCJQIjoiV2luMzIiLCJBTiI6Ik1haWwiLCJXVCI6Mn0%3D%7C3000%7C%7C%7C&sdata=4WZPHv6QwQIgpAq5AhdG8FUzlWPeiia548PAl7bMS5Y%3D&reserved=0).

Reviewer #1: No

[NOTE: If reviewer comments were submitted as an attachment file, they will be attached to this email and accessible via the submission site. Please log into your account, locate the manuscript record, and check for the action link "View Attachments". If this link does not appear, there are no attachment files.]

While revising your submission, please upload your figure files to the Preflight Analysis and Conversion Engine (PACE) digital diagnostic tool, [https://pacev2.apexcovantage.com/](https://eur02.safelinks.protection.outlook.com/?url=https%3A%2F%2Fpacev2.apexcovantage.com%2F&data=05%7C01%7Cmadj%40sund.ku.dk%7Cf279e3cbe12d453e184208dbcf560234%7Ca3927f91cda14696af898c9f1ceffa91%7C0%7C0%7C638331740540605517%7CUnknown%7CTWFpbGZsb3d8eyJWIjoiMC4wLjAwMDAiLCJQIjoiV2luMzIiLCJBTiI6Ik1haWwiLCJXVCI6Mn0%3D%7C3000%7C%7C%7C&sdata=N%2BqbNwIzuvsQx8nRcBhs%2F0EahPXli%2FVjgRjYkRzWmjQ%3D&reserved=0). PACE helps ensure that figures meet PLOS requirements. To use PACE, you must first register as a user. Registration is free. Then, login and navigate to the UPLOAD tab, where you will find detailed instructions on how to use the tool. If you encounter any issues or have any questions when using PACE, please email PLOS at [figures@plos.org](mailto:figures@plos.org). Please note that Supporting Information files do not need this step.
