# Supplementary material for: Mammography screening: Eliciting the voices of informed citizens
Source: PLoS One. 2025 Jan 9;20(1):e0317263. doi: 10.1371/journal.pone.0317263 (PMC11717236; doi:10.1371/journal.pone.0317263)
Supplement: Attachment — Submitted filename: Response to Reviewers.docx [file pone.0317263.s008.docx]

**Response to reviewers**

Journal Requirements:

Please review your reference list to ensure that it is complete and correct. If you have cited papers that have been retracted, please include the rationale for doing so in the manuscript text, or remove these references and replace them with relevant current references. Any changes to the reference list should be mentioned in the rebuttal letter that accompanies your revised manuscript. If you need to cite a retracted article, indicate the article’s retracted status in the References list and also include a citation and full reference for the retraction notice.

*Thank you for the comment. I have been through the reference list. WHO have moved their position paper to a new webpage which is now added to the reference list.*

Additional Editor Comments:

Dear Authors,

Thank you very much for the extensive revision!
Only a few points remain:

- In Methods, under questionnaire, please include the item formulation used to assess the participants' view on what they considered to be an acceptable mortality reduction from mammography screening.

*Thank you for the comment. In the revised manuscript we now mention the item regarding mortality reduction and the item regarding overdiagnosis in the methods section under questionnaire.*

- Table 1 should follow the PLOSONE guidelines and the font size be equal to the manuscript text. Some response options reported in Table 1 are put in one line, others in separate lines.

*Thank you for the comment. In the revised manuscript Table 1 have the same font size and each response option have a separate line.*

- On P. 10 (without mark-ups), from lines 6-7, there is a break.

*Thank you for the comment. There is no longer a break.*

- Regarding my concern about the robustness of the dominance analyses, and in the light of the significance of the correlation coefficents for change in knowledge index and change in recommendation, it might be more comprehensible for the reader to not communicate importance for items with non-significant correlations

*Thank you for the comment. Statistical significance is not the same as importance. Notably in small data sets, such as the one in the present paper, relatively large effects that may be considered important given the scenario at hand may find themselves not significant. Also, absence of evidence is not evidence of absence; but the reviewer/editor knows this. The findings in Table 3 that none of the knowledge items has a statistically significant association to “change in recommendation” may then serve as a warning that maybe the individual knowledge items are not very important factors to this outcome, that there are other factors that are better determinants for “change in recommendation” (which is not unreasonable to assert), or that not a single item, but rather several items determine the outcome. The logical premise of the relative importance approach is that IF we consider the effects of the items on an outcome important, WHAT is then the importance of these effects relative to each other (notably accounting for the intercorrelatedness of the items; something that is not taken into account in the marginal associations listed in Table 3). This will give an answer, even if none of the effects may be considered important in any absolute sense. All in all we think that the relative importance results give a good, and intuitive, indication of which items feed into the outcome most; the tables would be rather convoluted if we were to omit the relative importance for the non-significant results.”*
