# Supplementary material for: Mammography screening: Eliciting the voices of informed citizens
Source: PLoS One. 2025 Jan 9;20(1):e0317263. doi: 10.1371/journal.pone.0317263 (PMC11717236; doi:10.1371/journal.pone.0317263)
Supplement: Attachment — Submitted filename: Response to reviewers.docx [file pone.0317263.s009.docx]

Journal Requirements:

Please review your reference list to ensure that it is complete and correct. If you have cited papers that have been retracted, please include the rationale for doing so in the manuscript text, or remove these references and replace them with relevant current references. Any changes to the reference list should be mentioned in the rebuttal letter that accompanies your revised manuscript. If you need to cite a retracted article, indicate the article’s retracted status in the References list and also include a citation and full reference for the retraction notice.

*Thank you for the comment. I have been through the reference list again and I am not aware that I have cited papers that have been retracted. Please let me know if you are aware of any retractions and I will correct it at once.*

Additional Editor Comments:

Dear Authors,

In response to a discussion with both the editorial board and a fourth reviewer on the dominance analyses performed in your study, finally, the following information should be added:
- provide a lot more details regarding the procedure employed for their DA, "in particular the regression models [..][that] preceded the DAs" and the parameters used to determine the ranking of coefficients.
- The reviewer notices "whether the authors are referring to general, conditional, or complete".
My general point is the following: Please consider the update in terms of a reproducibility perspective from potential readers.

*Thank you for the comment. In the revised manuscript we describe the DA in more detail.*
